# Cd(II)/Mn(II)/Co(II)/Ni(II)/Zn(II) Coordination Polymers Built from Dicarboxylic Acid/Tetracarboxylic Acid Ligands: Their Structural Diversity and Fluorescence Properties

**DOI:** 10.3390/polym15071803

**Published:** 2023-04-06

**Authors:** Lu Liu, Jian-Min Li, Meng-Di Zhang, Hui-Jie Wang, Ying Li, Zhen-Bei Zhang, Zi-Fang Zhao, Yu Xi, Yuan-Yuan Huang, Jie Xu, Bo Zhang, Jun Chen, Cheng-Xing Cui

**Affiliations:** 1 School of Chemistry and Chemical Engineering, Henan Institute of Science and Technology, Xinxiang 453003, Chinajunchen713@126.com (J.C.); 2 School of Resources and Environment, Henan Institute of Science and Technology, Xinxiang 453003, China

**Keywords:** coordination complex, fluorescence properties, density functional theory

## Abstract

Six Cd(II)/Mn(II)/Co(II)/Ni(II)/Zn(II) coordination complexes are formulated as [Cd_2_(X^2−^)_2_(*μ*_3_-O)_2/3_]*_n_* (**1**), [Mn_2_(X^2−^)_2_(*μ*_3_-O)_2/3_]*_n_* (**2**), {[Co_1.5_(Y^4−^)_0.5_(4,4′-bpy)_1.5_(OH^−^)]·2H_2_O}*_n_* (**3**), {[Ni(X^2−^)(4,4′-bpy)(H_2_O)_2_]·4H_2_O}*_n_* (**4**), [Zn(*m*-bdc^2−^)(bebiyh)]_n_ (**5**), and [Cd(5-tbia^2−^)(bebiyh)]_n_ (**6**) (H_2_X = 3,3′-(2,3,5,6-tetramethyl-1,4-phenylene) dipropionic acid. H_4_Y = 2,2′-(2,3,5,6-tetramethyl-1,4-phenylene)bis(methylene) dimalonic acid, bebiyh = 1,6-bis(2-ethyl-1H-benzo[d]imidazol-1-yl)hexane, *m*-H_2_bdc = 1,3-benzenedicarboxylic acid, and 5-H_2_tbia = 5-(tert-butyl)isophthalic acid) were obtained by hydrothermal reactions and structurally characterized. Complexes **1** and **2** have a 6-connected 3D architecture and with several point symbols of (3^6^·4^6^·5^3^). Complex **3** features a 5-connected 3D net structure with a point symbol of (5·6^9^). Complex **4** possesses a 4-connected 2D net with a vertex symbol of (4^4^·6^2^). Complex **5** is a 3-connected 2D network with a point symbol of (6^3^). Complex **6** is a (3,3)-connected 2D network with a point symbol of (6^3^)_2_. In addition, complexes **1** and **4** present good photoluminescence behaviors. The electronic structures of **1** and **4** were investigated with the density functional theory (DFT) method to understand the photoluminescence behaviors.

## 1. Introduction

The research and development of materials play vital roles in the development of modern society [1,2,3,4]. As a new type of functional material, complexes’ structures are modifiable and thus have easy to modify functions [5,6,7]. In the field of materials chemistry, the complex has been a hot topic [8,9,10].

Structure determines performance. If you want to get the desired performance of the complex, you have to design it properly which is exactly what we have been trying to pursue [11,12,13]. Complexes are self-assembled by central metal ions or clusters (inorganic components) and organic ligands (organic components), so selecting appropriate metal ions and organic ligands can realize the design and construction of this material [14,15]. Among them, the variety of ligands is extremely large. The organic ligands with different configurations have an important influence on the synthesis and structure of complexes. In terms of the toughness of the ligand, ligands can be divided into rigid, flexible, or semirigid. Although the stability of the complex constructed by rigid ligands is good, rigid ligands cannot twist at will, which makes the structure of the complex monotonous. Although more complex, novel, and exotic sturctures with varied configurations can be obtained using flexible ligands, complex structures synthesized by the ligands are difficult to control. To our satisfaction, semirigid ligands have the characteristics of both rigid and flexible ligands.

To date, many polycarboxylic acid ligands have been employed to construct complexes due to the abundant coordination patterns of carboxylic acids [16,17,18,19,20,21,22]. Carboxylic acids have the following advantages: firstly, the O atom on the carboxylic acid group has a strong electron-donating ability and it is easy to coordinate with metal ions. Secondly, the coordination modes of carboxylic groups are flexible and varied. There are roughly three modes: single tooth, chelate, and bridge. When coordinating with more than one metal, the three types of double, three, and four teeth are displayed. In addition, the different orientations of coordination bonds between metal ions and O atoms can be expressed as cis–cis, cis–anti, and anti–anti patterns. The structural diversity of the coordination patterns of carboxylic acids is impressive. Thirdly, carboxyl groups are completely or partially deprotonated, rendering them hydrogen bond acceptors or hydrogen bond donors. In this way, hydrogen bonds can be formed with more electronegative atoms such as O, N, and F, thus contributing to the formation of a supramolecular structure. Fourthly, the conjugation property of the aromatic ring is conducive to electron transfer. Therefore, semirigid polycarboxylate ligands are our first choice followed by rigid ligands. In addition, the mixing strategy of the polycarboxylic acid and N-donor ligand is also an effective method in the synthesis of multi-dimensional structures [23,24,25,26,27,28].

**Scheme 1:**
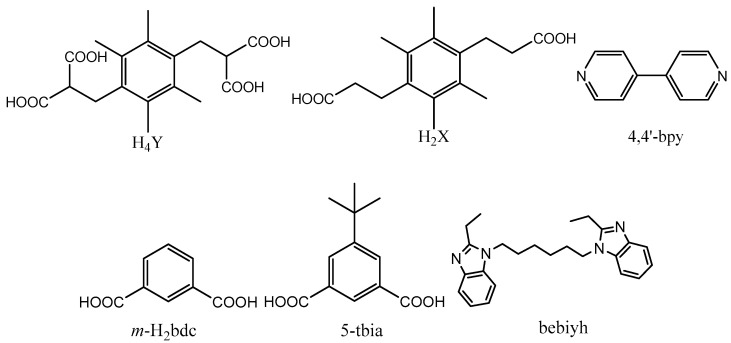
The organic ligand in this paper.

In view of this, the semirigid 3,3′-(2,3,5,6-tetramethyl-1,4-phenylene)dipropionic acid (H_2_X), 2,2′-(2,3,5,6-tetramethyl-1,4-phenylene)bis(methylene)dimalonic acid (H_4_Y), rigid 1,3-benzenedicarboxylic acid (*m*-H_2_bdc), and 5-(tert-butyl)isophthalic acid) (5-H_2_tbia) are selected as the primary ligand in this paper (Scheme 1). When 4,4′-bipyridine (4,4′-bpy) is present or not present, Cd(II) salt/Mn(II) salt/Co(II) salt/Ni(II) salt/Zn(II) salt reacts with H_2_X/H_4_Y/*m*-H_2_bdc/5-H_2_tbia to prepare six complexes: [Cd_2_(X^2−^)_2_(*μ*_3_-O)_2/3_]*_n_* (**1**), [Mn_2_(X^2−^)_2_(*μ*_3_-O)_2/3_]*_n_* (**2**), {[Co_1.5_(Y^4−^)_0.5_(4,4′-bpy)_1.5_(OH^−^)]·2H_2_O}*_n_* (**3**), {[Ni(X^2−^)(4,4′-bpy)(H_2_O)_2_]·4H_2_O}*_n_* (**4**), [Zn(*m*-bdc^2−^)(bebiyh)]_n_ (**5**), and [Cd(5-tbia^2−^)(bebiyh)]_n_ (**6**). We discuss the crystal structures of **1–6** and investigate the fluorescence properties of **1** and **4**.

## 2. Materials and Methods

All reagents and solvents were purchased commercially except for H_2_X and H_4_Y [29]. In the region of 400–4000 cm^−1^, FT-IR spectra were tested on an FTIR-7600 spectrophotometer. The C, H, and N content was recorded on a FLASH EA 1112 elemental analyzer. The luminescence properties were studied using a Cary Eclipse fluorescence spectrophotometer.

### 2.1. Synthesis

**Synthesis of [Cd_2_(X^2−^)_2_(*μ*_3_-O)_2/3_]*_n_* (1).** A hybrid of Cd(NO_3_)_2_·4H_2_O (0.0308 g), H_2_X (0.0138 g), DMF(4 mL), and H_2_O (2 mL) was placed in a 25 mL reactor. It was heated at 100 °C for three days. It was then cooled to produce a colorless crystal **1**. The yield is 36% (based on Cd). Anal. Calcd for C_96_H_120_Cd_6_O_26_ (%): C, 48.76, and H, 5.11. Found: C, 48.77, and H, 5.14. IR (KBr, cm^−1^): 3448(m), 2987(w), 1600(vs), 1428(m), 1319(w), 1226(vw), 1178(m), 1029(w), 1002(w), 946(w), 889(w), 786(w), 763(vw), 607(w), and 474(w).

**Synthesis of [Mn_2_(X^2−^)_2_(*μ*_3_-O)_2/3_]*_n_* (2).** A hybrid of MnCl_2_·4H_2_O (0.0297 g), H_2_X (0.0138 g), DMF (3 mL), EtOH (3 mL), and H_2_O (2 mL) was placed in a 25 mL reactor. It was also heated at 100 °C for three days. It was then cooled to produce a colorless crystal **2**. The yield is 25% (based on Mn). Anal. Calcd for C_96_H_120_Mn_6_O_26_ (%): C, 57.09, and H, 5.99. Found: C, 57.12, and H, 5.97. IR (KBr, cm^−1^): 3417(s), 2983(vw), 1706(vw), 1616(s), 1488(w), 1440(w), 1398(s), 1322(s), 1261(w), 1222(w), 1170(w), 1002(w), 939(w), 835(w), 759(w), 713(vw), 611(s), and 485(s).

**Synthesis of {[Co_1.5_(Y^4−^)_0.5_(4,4′-bpy)_1.5_(OH^−^)]·2H_2_O}*_n_* (3).** A hybrid of Co(NO_3_)_2_·6H_2_O (0.0291 g), H_4_Y (0.0180 g), 4,4′-bpy (0.0156 g), CH_3_CN (6 mL), and H_2_O (2 mL) was placed in a 25 mL reactor. It was heated at 95 °C for four days. It was then cooled to produce a purple rod crystal **3**. The yield is 10% (based on Co). Anal. Calcd for C_48_H_54_Co_3_N_6_O_14_ (%), C, 51.67; H, 4.88; and N, 7.53. Found: C, 51.64; H, 4.90; and N, 7.49.

**Synthesis of {[Ni(X^2−^)(4,4′-bpy)(H_2_O)_2_]·4H_2_O}*_n_* (4).** A hybrid of Ni(NO_3_)_2_·6H_2_O (0.0436 g), H_2_X (0.0138 g), 4,4′-bpy (0.0156 g), NaOH (0.008 g), and H_2_O (8 mL) was placed in a 25 mL reactor. It was heated at 130 °C for three days. It was then cooled to produce a green strip crystal **4**. The yield is 16% (based on Ni). Anal. Calcd for C_26_H_40_NiN_2_O_10_ (%), C, 52.10; H, 6.72; and N, 4.67. Found: C, 52.13; H, 6.70; and N, 4.64.

**Synthesis of [Zn(*m*-bdc^2−^)(bebiyh)]_n_ (5).** Zn(Ac)_2_·2H_2_O (0.2 mmol), bebiyh (0.1 mmol), *m*-H_2_bdc (0.2 mmol), NaOH (0.4 mmol), and H_2_O (8 mL) were mixed and heated in a 25-mL steel vessel at 120 °C for 3 days. After cooling the mixture, colorless crystals were obtained at a 12% yield (based on Zn).

**Synthesis of [Cd(5-tbia^2−^)(bebiyh)]_n_ (6).** Cd(NO_3_)_2_·4H_2_O (0.2 mmol), bebiyh (0.1 mmol), 5-H_2_tbia (0.2 mmol), NaOH (0.4 mmol), and H_2_O (8 mL) were mixed and heated in a 25-mL steel vessel at 170 °C for 3 days. After cooling the mixture, crystals of **6** were obtained at an 11% yield (based on Cd).

### 2.2. X-ray Crystallography

Crystallographic data for **1–6** were collected using an Xcalibur Eos Gemini CCD diffractometer (Mo-Kα, λ = 0.71073 Å). Absorption corrections were applied by using a multi-scan program. The data were corrected for Lorentz and polarization effects. Structures were solved by immediate methods and refined with a full-matrix least-squares technique based on *F*^2^ using the ShelXL software package [30]. Then, all of the non-hydrogen atoms were refined anisotropically. The hydrogen atoms of ligands were assigned at perfect positions capitalizing on a riding model and then they were refined isotropically [30]. Crystallographic crystal data and structure refinement details for **1–6** are summarized in Table S1, while selected bond lengths and bond angles for **1–6** are listed in Table S2.

## 3. Results and Discussion

### 3.1. Crystal Structure Description of Complexes ***1–6***

#### 3.1.1. Crystal Structures of [Cd_2_(X^2−^)_2_(μ_3_-O)_2/3_]_n_ (**1**) and [Mn_2_(X^2−^)_2_(μ_3_-O)_2/3_]_n_ (**2**)

Crystals **1** and **2** are isostructural. To be concise, only the structure of **1** is described in detail. The coordination environment of the Mn(II) ions and their correlation structure diagram in crystal **2** is presented in Figure S1. The asymmetric unit of **1** is composed of two Cd(II) atoms, two X^2−^, and 2/3 *μ*_3_-oxygen atoms. Each Cd1(II) atom has a hexagonal configuration formed by five carboxyl O atoms (O1, O2, O4, O5, and O5A) from five X^2−^ anions and one O3 atom from *μ*_3_-O. Cd1-O bond length is between 2.190(3) and 2.436(6) Å (Figure 2a). Each Cd2(II) atom has a hexagonal configuration formed by five carboxyl O atoms (O6B, O7, O8B, O10, and O10A) from five X^2−^ anions and one O9 atom from *μ*_3_-O. The Cd2-O bond length is between 2.210(6) and 2.451(6) Å. The O–Cd–O bond angles were in the range of 77.0(2)–169.3(2)°. In **2**, the Mn1(II) atom and Mn2(II) atom both adopt a six-coordinated configuration, respectively. The Mn1-O bond length is between 2.116(4) and 2.321(4) Å and the Mn2-O bond length is between 2.121(4) and 2.281(4) Å. The O–Mn–O bond angles were in the range of 77.27(15)–172.91(18)°.

**Figure 1:**
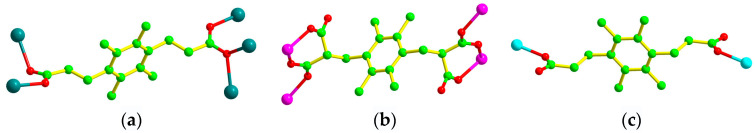
Coordination patterns of acids in complexes. (**a**) The coordination pattern of X^2−^ in **1**. (**b**) The coordination pattern of X^2−^ in **3**. (**c**) The coordination pattern of X^2−^ in **4**.

**Figure 2:**
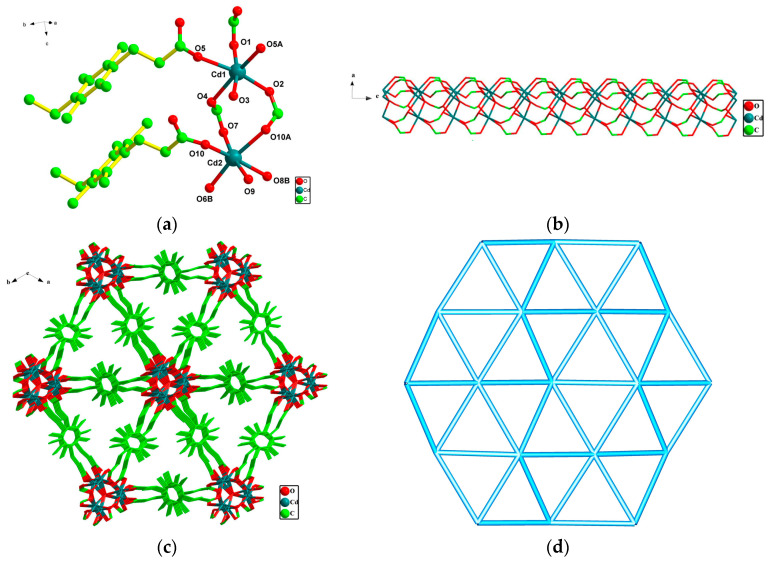
(**a**) Surrounding environment map of Cd(II) in complex **1**, symmetric opcode: A = 1 − y, x − y, z; B = x, y, 1 + z. (**b**) Cd(II)/^X2−^ 1D chain. (**c**) The 3D structure diagram of complex **1**. (**d**) Schematic view of the 3D topology network for **1**.

In **1**, the ligand X^2−^ exhibits a coordination pattern (Figure 1a). In this pattern, two carboxyl groups appear as *μ*_2_-*η*^1^:*η*^1^ and *μ*_3_-*η*^1^:*η*^2^, respectively, bridged with five Cd(II) ions. Based on this connection pattern, Cd1 and symmetrically related Cd1 atoms are bridged together by three carboxyl oxygen atoms and a *μ*_3_-O to produce a three-nucleated [Cd_3_O_4_] unit (SBU-A). The Cd2 atom and the symmetrically related Cd2 atom are also joined together by three carboxyl oxygen atoms and a *μ*_3_-O bridge to produce a three-nucleated [Cd_3_O_4_] unit (SBU-B). SBU-A and SBU-B are interchangeably connected by carboxyl oxygen atoms of X^2−^, resulting in a 1D chain structure (Figure 2b). The 1D chain forms a 3D structure under the extension of X^2−^ (Figure 2c).

Topologically, the [CdO] unit can be considered as a 6-connected node, which is connected to six equivalent nodes through six X^2−^ ligands. Each X^2−^ links two [CdO] units, so the X^2−^ can be simplified as links. Accordingly, the whole structure of **1** is related to a 6-connected network with a Schläfli symbol of (3^6^·4^6^·5^3^) topology (Figure 2d).

#### 3.1.2. Crystal Structures of {[Co_1.5_(Y^4−^)_0.5_(4,4′-bpy)_1.5_(OH^−^)]·2H_2_O}_n_ (**3**)

**Figure 3:**
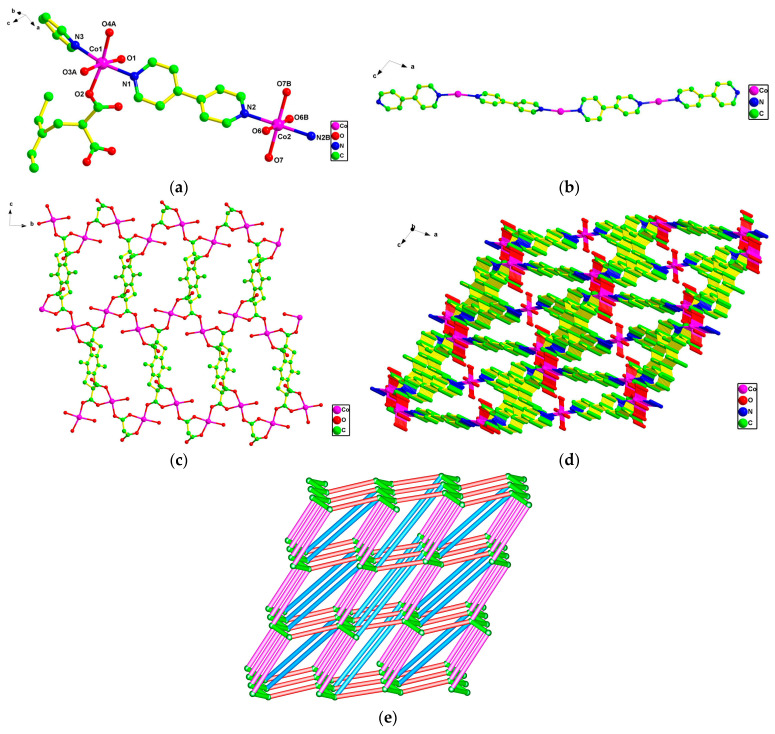
(**a**) Surrounding environment map of Co(II) in complex **3**, symmetric opcode: A = 0.5 − x, 0.5 + y, 0.5 − z; B = 1 − x, −y, −z. (**b**) Co(II)/4,4′-bpy 1D chain. (**c**) Co(II)/Y^4−^ 2D layer. (**d**) The 3D structure diagram of complex **3**. (**e**) Schematic view of the 3D topology network for **3**.

The asymmetric unit of **3** is composed of one and a half Co(II) atoms, half a Y^4−^ anion, one and a half 4,4′-bpy, a coordinated OH^−^, and two dissociative H_2_O molecules. Each Co1(II) atom has a hexagonal configuration formed by four carboxyl O atoms (O2, O3A, and O4A) from four Y^4−^ anions, one O1 atom from coordinated H_2_O molecules, and two N atoms (N1 and N3) from two separate 4,4′-bpy (Figure 3a). The Co1-O bond length is between 2.054(5) and 2.159(6) Å. The Co1-N bond length is between 2.170(6) and 2.184(6) Å. Each Co2(II) atom has a hexagonal configuration formed by four carboxyl O atoms (O6, O7, O6B, and O7B) and two N atoms (N2 and N2B). The Co2-O bond length is between 2.105(6) and 2.112(7) Å. The Co2-N bond lengths are all 2.176(7) Å. The O/N–Co–O/N bond angles were in the range of 81.6(2)–180.0(4)°.

In **3**, the 1D chain (Co(II)/4,4′-bpy chain) along the *b*-axis is also built by 4,4′-bpy ligands and Co(II) ions (Co1, symmetrically related Cd1 atoms and Co2) with a Co…Co distance of 11.4397 Å and 11.4606 Å (Figure 3b). The ligand Y^4−^ exhibits a coordination pattern (Figure 1b). In this pattern, four carboxyl groups appear as *μ*_4_-*η*^1^:*η*^2^:*η*^1^:*η*^2^, bridged with four Co(II) ions. Based on this connection pattern, Co1 and symmetrically related Cd1 atoms are bridged together by four carboxyl oxygen atoms to form a 2D layer (Figure 3c). The combination of 1D chains of Co(II)/4,4′-bpy and 2D Co(II)/Y^4−^ generates the 3D structure of **3** (Figure 3d).

As depicted in Figure 3e, topological analysis is performed on **3**. If the binuclear unit constituted by Co1 and symmetrically related Cd1 atoms is taken as a 5-connector, the Y^4−^ and Cd2 atoms can be defined as linkers, and the 3D framework of **3** can be classified as a 5-connected net with point symbol of (5·6^9^).

#### 3.1.3. Crystal Structures of {[Ni(X^2−^)(4,4′-bpy)(H_2_O)_2_]·4H_2_O}_n_ (**4**)

**Figure 4:**
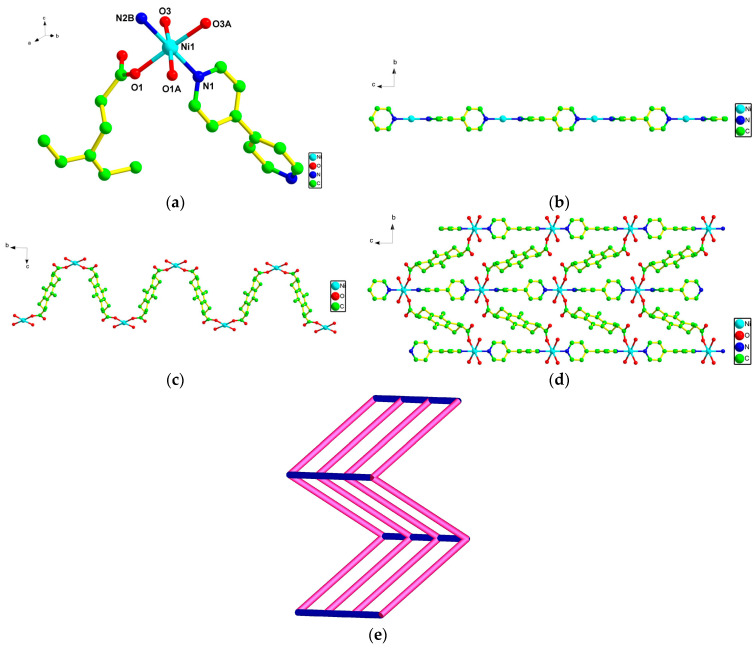
(**a**) Surrounding environment map of Ni(II) in complex **4**, symmetric opcode: A = x, 0.5 − y, z; B = 1 + x, y, 1 + z. (**b**) Ni(II)/X^2−^ 1D chain. (**c**) Ni(II)/4,4′-bpy 1D chain. (**d**) The 2D structure diagram of complex **4**. (**e**) Schematic view of the 2D topology network for **4**.

The asymmetric unit of **4** is composed of a Ni(II) atom, an X^2−^ anion, 4,4′-bpy, a coordinated H_2_O molecule, and four free H_2_O molecules. Each Ni(II) atom has a hexagonal configuration formed by two carboxyl O atoms (O1 and O1A) from two X^2−^ anions, two N atoms (N1, N2B) from two 4,4′-bpy ligands, and two O atoms (O3, O3A) from two coordinated H_2_O molecules (Figure 4a). The length of the Ni-O bond varies from 2.085(3)-2.086(3)/2.086(4) Å, while the length of the Ni-N1 bond is 2.117(5) Å, and the length of the Ni-N2B bond is 2.129(5) Å. The O/N–Ni–O/N bond angles were in the range of 81.6(2)–180.0(4)°.

X^2−^ adopts a trans-configuration and its two carboxyl groups adopt a single-tooth coordination mode (Figure 1c). The dihedral angle of the two pyridine rings of 4,4′-bpy is close to 90°. In **4**, 4,4′-bpy connects adjacent Ni(II) ions along an a-axis to generate a 1D straight chain structure (Figure 4b). Whereas, X^2−^ connects adjacent Ni(II) ions to generate a 1D wave-like chain structure along an a-axis (Figure 4c). Both 1D Ni(II)/4,4′-bpy chains and 1D Ni(II)/X^2−^ chains are alternately connected to form 2D layer structures (Figure 4d).

To further demonstrate the overall 2D structure of **4**, we can consider each Ni(II) as a 4-connecting node which is linked to four equivalent nodes through two X^2−^ anions and two 4,4′-bpy. X^2−^ and 4,4′-bpy are simplified as linear linkers separately. The whole structure of **4** can be simplified to a 4-connected net with a vertex symbol of (4^4^·6^2^) (Figure 4e).

#### 3.1.4. Crystal Structure of [Zn(m-bdc^2−^)(bebiyh)]_n_ (**5**)

Each asymmetric unit of **5** consists of one Zn(II) ion, one bebiyh, and one *m*-bdc^2−^. The Zn(II) ion adopts a four-coordinated configuration ligated by two nitrogen atoms (N1 and N3) from two bebiyh as well as two oxygen atoms (O2, O3A) from two *m*-bdc^2−^ anions (Figure S2a). The structural index parameter (*τ*_4_) [31,32] is close to 1.0, indicating that the geometry around Zn(II) can be described as a tetrahedral geometry. The Zn−O bond length is 1.980(8) Å and the Zn−N bond length is the range of 2.081(8)–2.093(10) Å. Bebiyh adopts a symmetric *trans*-conformation with N_donor_…N−C_sp3_…C_sp3_ torsion angles of 102.601° and 114.332°. In **5**, the two bebiyh act as a bidentate mode to joint two adjacent Zn(II) ions to form a 26-membered ring with a Zn···Zn separation of 11.3803 Å (Figure S2b). The *m*-bdc^2−^ adopted a *μ*_2_*-η*^1^*:η*^1^ mode. Each *m*-bdc^2−^ bridges two Zn(II) ions to generate a 1D Zn(II)/*m*-bdc^2−^ chain along the *c*-axis with a Zn···Zn separation of 10.4882 Å (Figure S2c). The combination of the 1D Zn(II)/*m*-bdc^2−^ chain and the 26-membered ring produces the 2D structure of **5** (Figure S2d) by sharing zinc ions.

To further demonstrate the overall 2D structure of **5**, we can consider each Zn(II) as a 3-connecting node which is linked to three equivalent nodes through two *m*-bdc^2−^ anions and one 26-membered ring. The *m*-bdc^2−^ and 26-membered ring are simplified as linear linkers separately. The whole structure of **5** can be simplified to a 3-connected net with a vertex symbol of (6^3^) (Figure S2e).

#### 3.1.5. Crystal Structure of [Cd(5-tbia^2−^)(bebiyh)]_n_ (**6**)

Each asymmetric unit of **6** consists of one Cd(II) ion, one bebiyh, and one 5-tbia^2−^. The Cd1 ion adopts a five-coordinated configuration ligated by one nitrogen atom (N1) from two bebiyh as well as three oxygen atoms (O1A, O2A, and O3) from two 5-tbia^2−^ (Figure S3a). The structural index parameter (*τ*_5_) [31,32] is close to 0.1, indicating that the geometry around Cd1 can be described as a square pyramidal structure. The Cd1−O/N bond length is in the range of 2.191(6)–2.581(7) Å. Whereas, the O/N–Cd1–O/N bond angles were in the range of 53.2(2)–144.3(3)°. The Cd2 ion adopts a six-coordinated configuration ligated by two nitrogen atoms (N5 and N7) from two bebiyh as well as four oxygen atoms (O5, O6, O7, and O8) from two 5-tbia (Figure S3a). The Cd2−O/N bond length is in the range of 2.218(6)–2.607(6) Å. Whereas, O/N–Cd2–O/N bond angles were in the range of 53.8(2)–150.6(2)°.

In **6**, bebiyh adopts a symmetric *trans*-conformation with the N_donor_…N−C_sp3_…C_sp3_ torsion angle of 85.233° and 91.397°. The bebiyh act in bidentate mode to join two adjacent Cd(II) ions to form a 1D Cd(II)/bebiyh chain along the *a*-axis with a Cd···Cd separation of 8.9479 and 13.1222 Å (Figure S3b). The 5-tbia^2−^ adopted a *μ*_2_*-η*^1^*:η*^1^ mode. Each 5-tbia bridges two Cd(II) ions to generate a 1D Cd(II)/5-tbia chain with a Cd…Cd separation of 8.9479 and 9.3580 Å (Figure S3c). The combination of the 1D Cd(II)/bebiyh chain and Cd(II)/5-tbia^2−^ chain by sharing cadmium ions produces the 2D structure of **6** (Figure S3d).

To further demonstrate the overall 2D structure of **6**, we can consider each Cd (II) (Cd1(II) and Cd2(II)) as a 3-connecting node. The 5-tbia^2−^ and bebiyh are simplified as linear linkers separately. The whole structure of **6** can be simplified to a (3,3)-connected net with a vertex symbol of (6^3^)_2_ (Figure S3e).

### 3.2. Photoluminescence Properties

**Figure 5:**
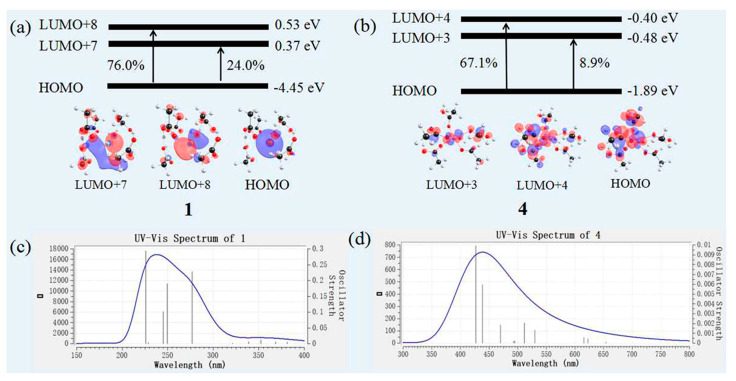
The calculated excitation of complexes **1** (**a**) and **4** (**b**) and the calculated emission spectrum of **1** (**c**) and **4** (**d**) at the theoretical level of TD−M06L/6−31G(d,p), where the SDD effective core potential was applied for the metallic elements.

We investigated the fluorescence spectrum of complexes **1** and **4** and the free ligand H_2_X (Figure S4). H_2_X shows an emission band at 300 nm (λ_ex_ = 282 nm). The emission band of 4,4′-bpy is 428 nm (λ_ex_ = 350 nm) [33]. The fluorescence emission peaks were observed at 301 nm for complex **1** (λ_ex_ = 281 nm) and 306 nm for complex **4** (λ_ex_ = 265 nm), respectively. The emission peak of **1** is similar to that of H_2_X, which may be mainly attributed to the endoligand emission of H_2_X. The emission band of complex **4** is red-shifted by 6 nm, thus corresponding to the emission band of H_2_X. As for 4,4′-bpy, the emission band of complex **4** is blue-shifted by 122 nm. This may be due to coordination with metal ions. To better understand the photoluminescent properties of complexes **1** and **4**, we further performed theoretical investigations on their model systems as shown in Figure 5. We optimized the four geometries at the theoretical level of M06L/6-31G(d,p) under a vacuum, where the SDD effective core potential was applied for the metallic elements. We further calculated the excited properties with the time dependent density function theory (TDDFT) method [34], where the option of nstates for TDDFT was set as 10 [35]. The calculated Cd2-O bond length is 2.558 Å, which is similar to the experimental results (between 2.210(6) and 2.451(6) Å). The calculated emission wavelength of complex **1** is 278 nm and the oscillator strength is as large as 0.228, which is consistent with the experimental results. Moreover, the relevant orbitals for the excited process are HOMO, LUMO+7, and LUMO+8, with corresponding energies of −4.45 eV, 0.37 eV, and 0.53 eV, respectively (Figure 5a). The luminescent processes are related to the frontier orbitals including HOMO, LUMO+3, and LUMO+4. For complex **4**, as shown in Figure 5b, the calculated luminescent properties are both relevant to the metal center, which is indicative of their crucial roles. As shown in Figure 5c,d where we gave the calculated emission spectrum of complexes **1** and **4**, the oscillator strength of **1** is arguably larger than **4**. This indicates that the emission of **4** is weaker than **1**, which is in line with the experimental observations.

## 4. Conclusions

Six new Cd(II)/Mn(II)/Co(II)/Ni(II)/Zn(II)-containing coordination complexes based on the dicarboxylic acid/tetracarboxylic acid ligands were synthesized. Complexes **1**, **2,** and **3** feature several 3D net structures. Complex **4**, **5**, **6** possesses a 2D layer structure, severally. The structure of the ligand has an important effect on the configuration of the complex, leading to the formation of different beautiful topologies. The theoretical calculation results indicate that the luminescence could be mainly related to the metal center for complexes **1** and **4**, while the oscillator strength of **1** is larger than **4**.

## Data Availability

Crystallographic data for **1–6** were deposited at the Cambridge Crystallographic Data Centre with CCDC reference numbers 2232253–2232256 and 2237514–2237515.

## Supplementary Materials

The following supporting information can be downloaded at: https://www.mdpi.com/article/10.3390/polym15071803/s1. Figure S1: (a) Surrounding environment map of Mn(II) in complex **2**, symmetric opcode: A = x, y, 1 + z; B = −y, x − y, 1 + z; C = 0.33333 − x + y, 0.66667 − x, − 0.33333 + z; (b) Mn(II)/X^2−^ 1D chain; and (c) the 3D structure diagram of complex **2**. Figure S2: (a) Coordination environment diagram around the Zn(II) center in **5**; (b) the 26-membered rings constructed by two bebiyh ligands and two Zn atoms; (c) 1D Zn/m-H_2_bdc chain; (d) the 2D layer structure of **5**; and (e) schematic view of the 2D topology network for **5**. Figure S3: (a) coordination environment diagram around the Cd(II) center in **6**; (b) 1D Cd/bebiyh chain; (c) 1D Cd/5-tbia chain; (d) 2D layer structure of **6**; and (e) schematic view of the 2D topology network for **6**. Figure S4: Photoluminescent emission spectrum of the free H_2_X ligand, complexes **1** and **4**. Figure S5: Experimental (red) and simulated (black) PXRD patterns of complex **1** (a), **2** (b), and **4** (c). Table S1: Crystallographic data and structure refinement details for complex **1–6**^a,b^. Table S2: Table S2 Selected Bond Lengths (Å) and Bond Angles (deg) for **1–6**^a^.

## Sample Availability

Sample of the compound is available from the authors.
