# Supplementary material for: Cd(II)/Mn(II)/Co(II)/Ni(II)/Zn(II) Coordination Polymers Built from Dicarboxylic Acid/Tetracarboxylic Acid Ligands: Their Structural Diversity and Fluorescence Properties
Source: Polymers (Basel). 2023 Apr 6;15(7):1803. doi: 10.3390/polym15071803 (PMC10098927; doi:10.3390/polym15071803)
Supplement: Supplementary file 1 [file polymers-15-01803-s001.zip › polymers-2181009-supplementary.pdf]

Cd(II)/Mn(II)/Co(II)/Ni(II)/Zn(II) Coordination Polymers Built from Dicarboxylic acid/Tetracarboxylic Acid Ligands: Their Structural diversity and Fluorescence properties

Lu Liu[1], Jian-Min Li[2], Meng-Di Zhang[1], Hui-Jie Wang[1], Ying Li[1], Zhen-Bei Zhang[1], Zi-Fang Zhao[1], Yu Xi[1], Yuan-Yuan Huang[1], Jie Xu[1], Bo Zhang[1], Jun Chen[1] and Cheng-Xing Cui[1,*]

[1] *School of Chemistry and Chemical Engineering, Henan Institute of Science and Technology, Xinxiang, Henan, 453003, P. R. China*
[2] *School of Resources and Environment, Henan Institute of Science and Technology, Xinxiang, Henan, 453003, P. R. China*
[*] Correspondence: chengxingcui@hist.edu.cn

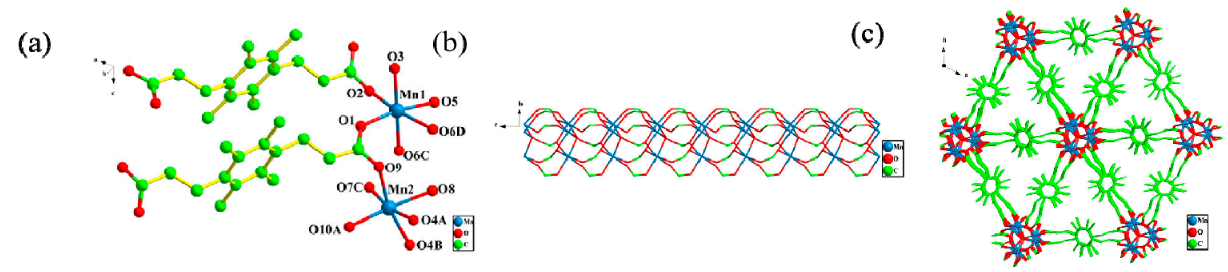

Figure S1 (a) Surrounding environment map of Mn(II) in complex **2**, symmetric opcode: A = x, y, 1 + z; B = -y, x - y, 1 + z; C = 0.33333 - x + y, 0.66667 - x, - 0.33333 + z; (b) Mn(II)/X^{2-} 1D chain; (c) The 3D structure diagram of complex **2**.

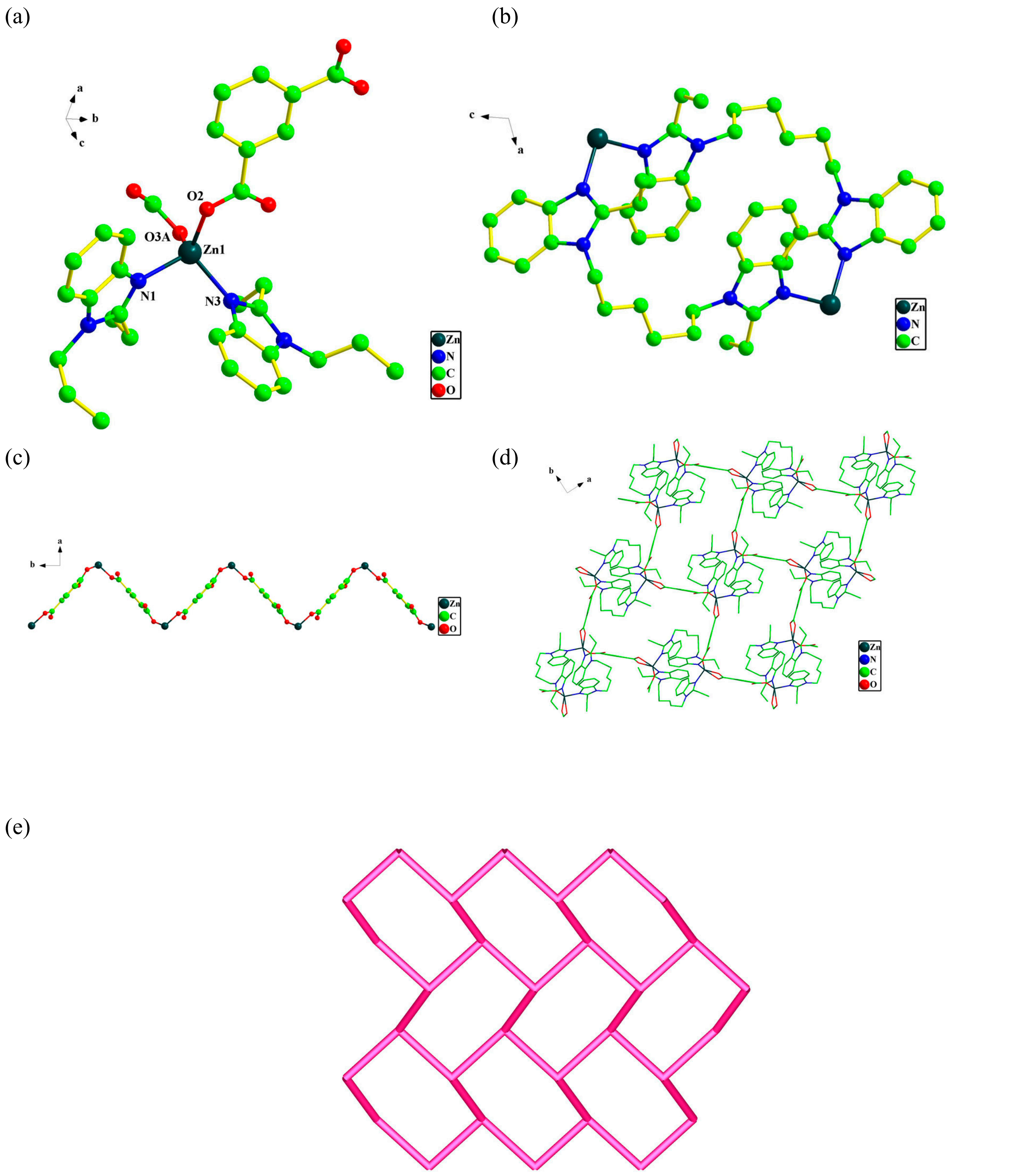

Figure S2 (a) Coordination environment diagram around the Zn(II) center in **5**. (b) The 26-membered rings constructed by two bebiyh ligands and two Zn atoms. (c) 1D Zn/*m*-H_2bdc chain. (d) 2D layer structure of **5**. (e) Schemetic view of the 2D topology network for **5**.

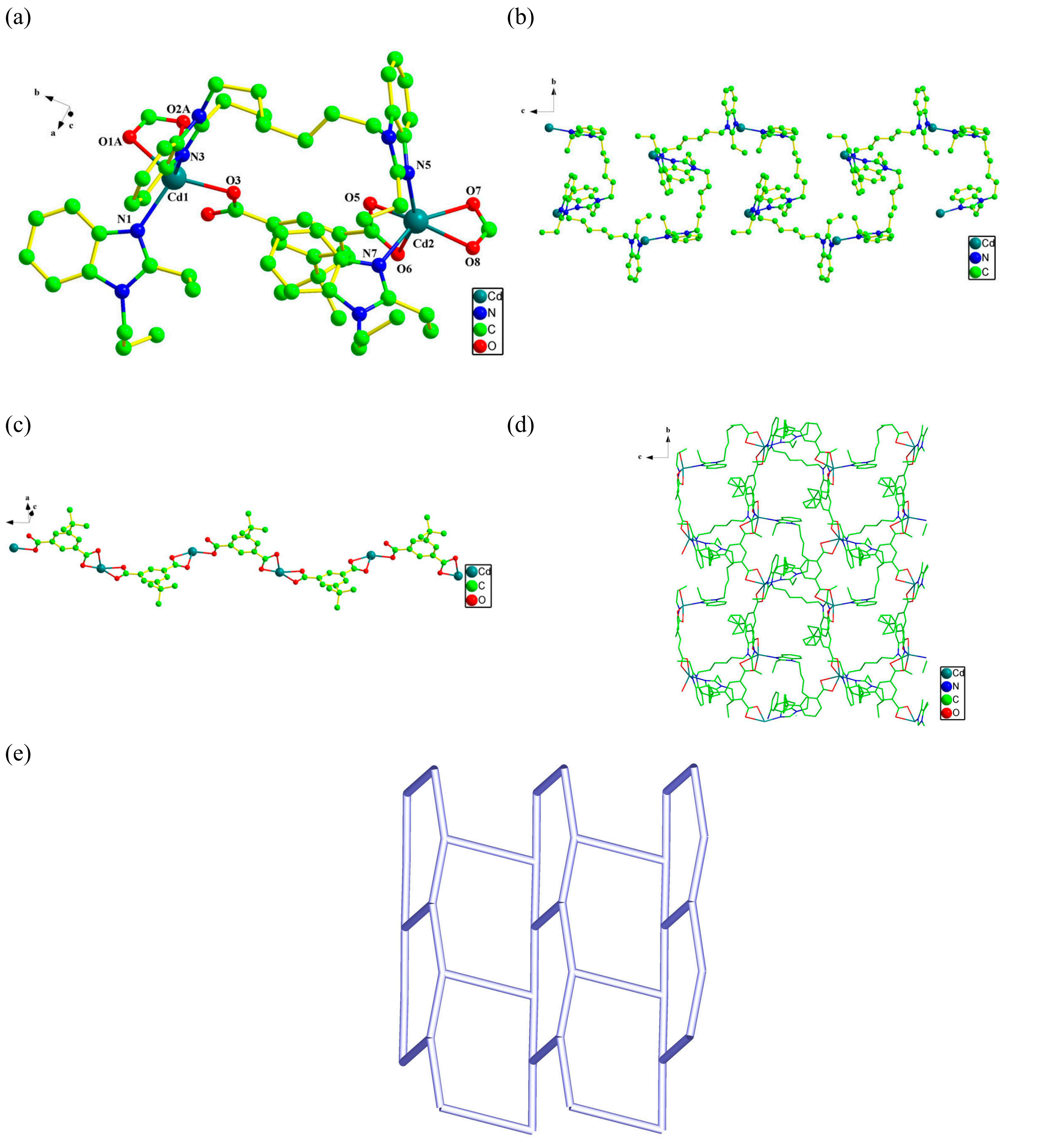

Figure S3 (a) Coordination environment diagram around the Cd(II) center in **6**. (b) 1D Cd/bebiyh chain. (c) 1D Cd/5-tbia chain. (d) 2D layer structure of **6**. (e) Schemetic view of the 2D topology network for **6**.

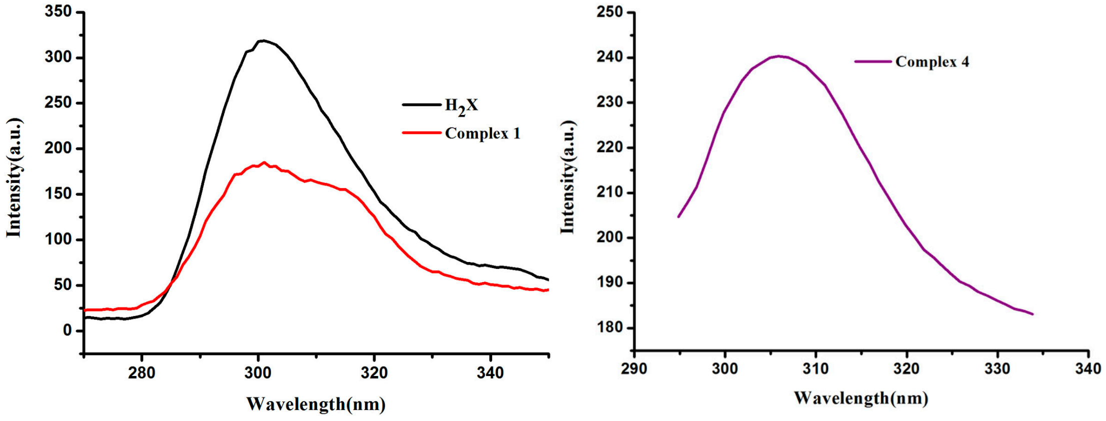

Figure S4 Photoluminescent emission spectrum of the free H_2X ligand, complex **1** and **4**.

PXRD analysis of complex 1, 2 and 4

PXRD analysis was performed to check the purity of **1, 2** and **4** (Figure S3). The peak positions of the as-synthesized sample were aligned with those simulated.

(a)

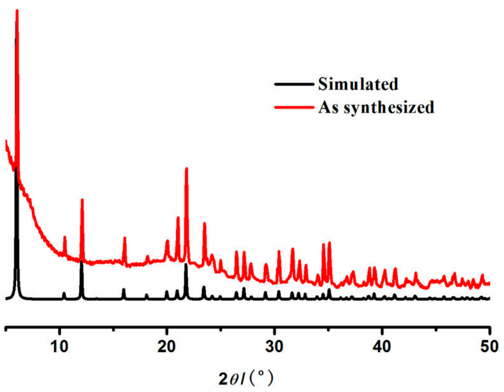

(b)

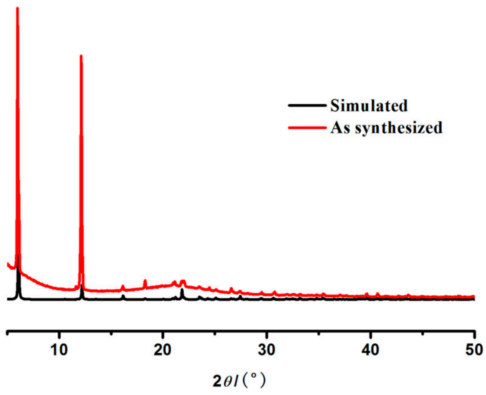

(c)

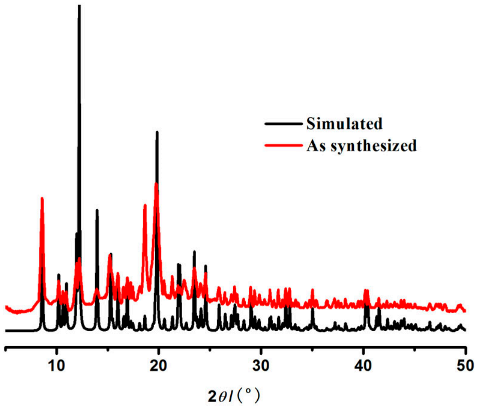

Figure S5 Experimental (red) and simulated (black) PXRD patterns of complex **1**(a), **2**(b) and **4**(c).

Table S1 Crystallographic data and structure refinement details for complex 1-6[a,b]

Complex	**1**	**2**	**3**	**4**	**5**	**6**
formula	$C_{96}H_{120}Cd_6O_{26}$	$C_{96}H_{120}Mn_6O_{26}$	$C_{48}H_{54}Co_3N_6O_{14}$	$C_{26}H_{40}N_2NiO_{10}$	$C_{32}H_{34}N_4O_4Zn$	$C_{72}H_{84}Cd_2N_8O_8$
fw	2364.32	2019.56	1115.76	599.29	604.00	1414.27
T/K	293(2)	293(2)	293(2)	293(2)	293(2)	293(2)
λ (Mo K), Å	0.71073	0.71073	0.71073	0.71073	0.71073	0.71073
Cryst syst	Hexagonal	Rhombohedral	Monoclinic	Monoclinic	Monoclinic	Orthorhombic
Space group	*R3*	*R3*	*C2/c*	*P21/m*	*P121/c1*	*Pna2(1)*
a (Å)	29.341(4)	28.993(4)	26.328(7)	8.9658(18)	12.670(3)	19.083(4)
b (Å)	29.341(4)	28.993(4)	8.7111(17)	16.111(3)	14.144(3)	17.919(4)
c (Å)	9.0195(18)	9.000(15)	25.620(8)	11.056(2)	18.264(4)	19.620(4)
α(°)	90	90	90	90	90	90
β(°)	90	90	108.95(3)	111.80(3)	109.92(3)	90
γ(°)	120	120	90	90	90	90
V ($Å^3$)	6724.6(19)	6552(11)	5557(3)	1482.8(5)	3077.4(12)	6709(2)
Z	3	3	4	2	4	4
$D_{calcd.}$(g·cm^{-3})	1.751	1.536	1.334	1.342	1.304	1.400
abs coeff/mm^{-1}	1.477	0.922	0.949	0.709	0.839	0.695
F(000)	3576	3162	2308	636	1264	2928
$\theta/2\theta$ (°)	1.39-27.92(θ)	1.40-27.91	2.48-25.50	2.35-25.49	1.710-23.050(θ)	1.539-25.000(θ)
GOF	1.069	0.985	0.990	1.043	1.077	1.031
R_1(I>2sigma(I))[a]	0.0581	0.0635	0.0986	0.0872	0.1187	0.0482
wR_2(I>2sigma(I))[b]	0.1288	0.1599	0.2293	0.1804	0.3448	0.1235

$^aR_1 = \sum||F_o|-|F_c||/\sum|F_o|$. $^bwR_2 = [\sum w(F_o^2 - F_c^2)^2/\sum w(F_o^2)^2]^{1/2}$.

Table S2 Selected Bond Lengths (Å) and Bond Angles (deg) for 1-6[a]

Complex **1**					
Cd(1)-O(3)	2.190(3)	Cd(1)-O(1)	2.194(6)	Cd(1)-O(2)	2.247(5)
Cd(1)-O(5)#2	2.384(6)	Cd(1)-O(4)	2.419(6)	Cd(1)-O(5)	2.436(6)
Cd(2)-O(7)	2.210(6)	Cd(2)-O(6)#3	2.219(6)	Cd(2)-O(9)	2.219(3)
Cd(2)-O(10)	2.396(6)	Cd(2)-O(10)#2	2.400(6)	Cd(2)-O(8)#3	2.451(6)
O(3)-Cd(1)-O(1)	168.5(2)	O(3)-Cd(1)-O(2)	101.4(2)	O(1)-Cd(1)-O(2)	89.6(2)
O(3)-Cd(1)-O(5)#2	78.1(2)	O(1)-Cd(1)-O(5)#2	103.3(2)	O(2)-Cd(1)-O(5)#2	100.5(2)
O(3)-Cd(1)-O(4)	89.1(2)	O(1)-Cd(1)-O(4)	89.0(2)	O(2)-Cd(1)-O(4)	83.0(2)
O(5)#2-Cd(1)-O(4)	167.17(19)	O(3)-Cd(1)-O(5)	77.0(2)	O(1)-Cd(1)-O(5)	91.5(2)
O(2)-Cd(1)-O(5)	169.3(2)	O(5)#2-Cd(1)-O(5)	89.6(3)	O(4)-Cd(1)-O(5)	86.3(2)
O(7)-Cd(2)-O(6)#3	88.8(2)	O(7)-Cd(2)-O(9)	170.9(2)	O(6)#3-Cd(2)-O(9)	99.9(2)
O(7)-Cd(2)-O(10)	103.5(2)	O(9)-Cd(2)-O(10)	77.9(2)	O(6)#3-Cd(2)-O(10)#2	169.2(2)
O(6)#3-Cd(2)-O(10)	99.7(2)	O(7)-Cd(2)-O(10)#2	93.2(2)	O(9)-Cd(2)-O(10)#2	77.8(2)
O(10)-Cd(2)-O(10)#2	90.1(3)	O(7)-Cd(2)-O(8)#3	89.0(2)	O(6)#3-Cd(2)-O(8)#3	83.7(2)
O(9)-Cd(2)-O(8)#3	89.20(19)	O(10)-Cd(2)-O(8)#3	167.01(19)	O(10)#2-Cd(2)-O(8)#3	85.77(19)
Complex **2**					
Mn(1)-O(1)	2.116(4)	Mn(1)-O(5)	2.153(3)	Mn(1)-O(3)	2.160(4)
Mn(1)-O(2)	2.220(4)	Mn(1)-O(6)#1	2.277(4)	Mn(1)-O(6)#2	2.321(4)
Mn(2)-O(10)#3	2.121(4)	Mn(2)-O(7)#2	2.137(4)	Mn(2)-O(8)	2.218(3)
Mn(2)-O(9)	2.244(4)	Mn(2)-O(4)#4	2.263(4)	Mn(2)-O(4)#3	2.281(4)
O(1)-Mn(1)-O(5)	169.83(17)	O(1)-Mn(1)-O(3)	93.73(16)	O(5)-Mn(1)-O(3)	96.38(17)
O(1)-Mn(1)-O(2)	92.74(15)	O(5)-Mn(1)-O(2)	89.36(14)	O(3)-Mn(1)-O(2)	84.55(16)
O(1)-Mn(1)-O(6)#1	99.15(15)	O(5)-Mn(1)-O(6)#1	78.24(15)	O(3)-Mn(1)-O(6)#1	98.57(15)
O(2)-Mn(1)-O(6)#1	167.45(13)	O(3)-Mn(1)-O(6)#2	168.56(13)	O(10)#3-Mn(2)-O(7)#2	92.83(17)
O(1)-Mn(1)-O(6)#2	92.95(15)	O(2)-Mn(1)-O(6)#2	85.87(15)	O(10)#3-Mn(2)-O(8)	172.91(18)
O(5)-Mn(1)-O(6)#2	77.27(15)	O(6)#1-Mn(1)-O(6)#2	89.52(19)	O(7)#2-Mn(2)-O(8)	94.25(17)
O(10)#3-Mn(2)-O(9)	92.87(16)	O(7)#2-Mn(2)-O(4)#4	98.04(15)	O(7)#2-Mn(2)-O(4)#3	167.71(13)
O(7)#2-Mn(2)-O(9)	84.65(15)	O(8)-Mn(2)-O(4)#4	78.07(15)	O(8)-Mn(2)-O(4)#3	77.70(16)
O(8)-Mn(2)-O(9)	87.97(14)	O(9)-Mn(2)-O(4)#4	165.92(13)	O(9)-Mn(2)-O(4)#3	85.79(15)
O(10)#3-Mn(2)-O(4)#4	100.76(15)	O(10)#3-Mn(2)-O(4)#3	95.34(16)	O(4)#4-Mn(2)-O(4)#3	89.44(19)
Complex **3**					
Co(1)-O(3)#1	2.054(5)	Co(1)-N(3)	2.170(6)	Co(2)-O(7)#2	2.105(6)
Co(1)-O(2)	2.084(5)	Co(1)-N(1)	2.184(6)	Co(2)-O(7)	2.105(6)
Co(1)-O(4)#1	2.124(5)	Co(2)-O(6)#2	2.112(7)	Co(2)-N(2)	2.176(7)
Co(1)-O(1)	2.159(6)	Co(2)-O(6)	2.112(7)	Co(2)-N(2)#2	2.176(7)
O(3)#1-Co(1)-O(2)	82.3(2)	O(4)#1-Co(1)-O(1)	100.6(2)	O(3)#1-Co(1)-N(1)	95.7(2)
O(3)#1-Co(1)-O(4)#1	85.9(2)	O(3)#1-Co(1)-N(3)	96.2(2)	O(2)-Co(1)-N(1)	96.4(2)
O(2)-Co(1)-O(4)#1	167.6(2)	O(2)-Co(1)-N(3)	91.2(2)	O(4)#1-Co(1)-N(1)	88.6(2)
O(3)#1-Co(1)-O(1)	172.8(3)	O(4)#1-Co(1)-N(3)	86.2(2)	O(1)-Co(1)-N(1)	81.6(2)
O(2)-Co(1)-O(1)	91.3(3)	O(1)-Co(1)-N(3)	87.2(2)	N(3)-Co(1)-N(1)	166.6(3)

O(6)#2-Co(2)-O(6)	180.0(4)	O(6)#2-Co(2)-O(7)#2	91.8(3)	O(6)-Co(2)-N(2)	89.7(3)
O(6)#2-Co(2)-O(7)	88.2(3)	O(6)-Co(2)-O(7)	91.8(3)	O(7)#2-Co(2)-N(2)	91.4(3)
O(7)#2-Co(2)-O(7)	180.0(6)	O(6)#2-Co(2)-N(2)	90.3(3)	O(7)-Co(2)-N(2)	88.6(3)
O(6)#2-Co(2)-N(2)#2	89.7(3)	O(6)-Co(2)-N(2)#2	90.3(3)	O(7)#2-Co(2)-N(2)#2	88.6(3)
O(7)-Co(2)-N(2)#2	91.4(3)	N(2)-Co(2)-N(2)#2	180.0(3)		
Complex **4**					
Ni(1)-O(1)#1	2.085(3)	Ni(1)-O(1)	2.085(3)	Ni(1)-O(3)	2.086(4)
Ni(1)-N(2)#2	2.129(5)	Ni(1)-N(1)	2.117(5)	Ni(1)-O(3)#1	2.086(3)
O(1)#1-Ni(1)-O(1)	84.88(19)	O(1)#1-Ni(1)-O(3)	175.33(14)	O(3)-Ni(1)-N(1)	91.55(15)
O(1)-Ni(1)-O(3)	90.45(14)	O(1)#1-Ni(1)-O(3)#1	90.45(14)	O(3)#1-Ni(1)-N(1)	91.55(15)
O(1)-Ni(1)-O(3)#1	175.33(14)	O(3)-Ni(1)-O(3)#1	94.2(2)	O(1)#1-Ni(1)-N(2)#2	91.23(15)
O(1)#1-Ni(1)-N(1)	88.47(15)	O(1)-Ni(1)-N(1)	88.47(15)	O(1)-Ni(1)-N(2)#2	91.23(15)
O(3)-Ni(1)-N(2)#2	88.73(14)	O(3)#1-Ni(1)-N(2)#2	88.73(14)	N(1)-Ni(1)-N(2)#2	179.6(2)
Complex **5**					
Zn(1)-N(1)	2.093(10)	Zn(1)-N(3)	2.081(8)	Zn(1)-O(2)	1.980(8)
Zn(1)-O(3)#1	1.980(8)	N(3)-Zn(1)-N(1)	101.8(4)	O(2)-Zn(1)-N(1)	106.5(4)
O(2)-Zn(1)-N(3)	112.7(4)	O(3)#1-Zn(1)-N(1)	112.4(3)	O(3)#1-Zn(1)-N(3)	108.0(3)
O(3)#1-Zn(1)-O(2)	114.8(4)				
Complex **6**					
Cd(1)-N(1)	2.300(7)	Cd(1)-N(3)	2.308(8)	Cd(1)-O(1)#1	2.245(6)
Cd(1)-O(2)#1	2.581(7)	Cd(1)-O(3)	2.191(6)	Cd(2)-N(5)	2.313(7)
Cd(2)-N(7)	2.311(7)	Cd(2)-O(5)	2.218(6)	Cd(2)-O(6)	2.607(6)
Cd(2)-O(7)	2.495(6)	Cd(2)-O(8)	2.242(7)	N(1)-Cd(1)-N(3)	106.3(3)
N(1)-Cd(1)-O(2)#1	138.8(3)	N(3)-Cd(1)-O(2)#1	108.1(3)	O(1)#1-Cd(1)-N(1)	99.1(2)
O(1)#1-Cd(1)-N(3)	99.8(3)	O(1)#1-Cd(1)-O(2)#1	53.2(2)	O(3)-Cd(1)-N(1)	110.0(3)
O(3)-Cd(1)-N(3)	91.6(3)	O(3)-Cd(1)-O(1)#1	144.3(3)	O(3)-Cd(1)-O(2)#1	91.2(2)
N(5)-Cd(2)-O(6)	150.2(2)	N(5)-Cd(2)-O(7)	106.4(2)	N(7)-Cd(2)-N(5)	96.8(3)
N(7)-Cd(2)-O(6)	87.3(2)	N(7)-Cd(2)-O(7)	150.6(2)	O(5)-Cd(2)-N(5)	96.5(2)
O(5)-Cd(2)-N(7)	93.5(2)	O(5)-Cd(2)-O(6)	53.8(2)	O(5)-Cd(2)-O(7)	101.3(2)
O(5)-Cd(2)-O(8)	146.6(3)	O(7)-Cd(2)-O(6)	81.4(2)	O(8)-Cd(2)-N(5)	111.7(3)
O(8)-Cd(2)-N(7)	100.2(3)	O(8)-Cd(2)-O(6)	96.4(3)	O(8)-Cd(2)-O(7)	54.8(2)

[a] Symmetry transformations used to generate equivalent atoms in complex (**1**): #2 -y+1,x-y,z; #3 x,y,z+1. (**2**) #1 -y+1/3,x-y-1/3,z-1/3; #2 -x+y+1/3,-x+2/3,z-1/3; #3 x,y,z+1; #4 -y,x-y,z+1; #5 -x+y,-x,z-1. (**3**) #1 -x+1/2,y+1/2,-z+1/2; #2 -x+1,-y,-z; (**4**): #1 x,-y+1/2,z; #2 x+1,y,z+1. (**5**): #1 -x+1,y-1/2,-z+1/2. (**6**): #1 x,y+1,z.
